# Successful use of tocilizumab and casirivimab/imdevimab in a twin pregnancy with critical COVID-19 – A case report

**DOI:** 10.1016/j.heliyon.2024.e31737

**Published:** 2024-05-22

**Authors:** Nina Grasselli Kmet, Miha Lučovnik, Matjaž Jereb, Kristina Nadrah

**Affiliations:** a Infectious Diseases Department, University Medical Centre, Ljubljana, Slovenia; b Faculty of Medicine, University of Ljubljana, Slovenia; c Division of Gynaecology and Obstetrics, University Medical Centre, Ljubljana, Slovenia

**Keywords:** Twin pregnancy, Critical COVID-19, Tocilizumab, Casirivimab/imdevimab

## Abstract

COVID-19 in pregnancy is associated with increased maternal morbidity and mortality as well as higher risk for hospitalization in intensive care unit and mechanical ventilation. We present a 38-year-old 21^+5^week pregnant unvaccinated woman with twins and critical COVID-19 pneumonia caused by Delta SARS-CoV-2 strain. Because of rapid worsening of respiratory condition despite standard of care treatment with steroids, she received a combination of casirivimab/imdevimab and tocilizumab. After therapy we noticed respiratory improvement and after 10 days she was extubated. Due to selective fetal growth restriction of one of the twins, a planned caesarean section was performed at 34^+6^ weeks. Presented case indicates favorable outcome and safe use of casirivimab/imdevimab and tocilizumab in critical COVID-19, as no severe or minor signs or symptoms in the case presentation were observed neither in the mother nor in infants during the time of observation.

## 1 Introduction

COVID-19 in pregnancy is associated with higher risk of preeclampsia/eclampsia, preterm delivery, severe neonatal morbidity and maternal mortality. Higher maternal age and comorbidities, especially obesity, pregestational diabetes and chronic lung disease, have been shown to increase risk of severe/critical COVID-19, and adverse pregnancy outcome. Higher rate of cesarean sections (CS) was observed, as well as preterm deliveries [[1], [2], [3]]. Corticosteroids, which were shown to reduce mortality, are used as a standard of care (SoC) in pregnancy for critical COVID-19 [4]. Although addition of tocilizumab was shown to improve survival and shorten the time to clinical improvement [5], there is paucity of data on safety and efficacy of its use in pregnancy [6].

### 1.1 Case presentation

A 38-year-old pregnant woman who was not vaccinated against COVID-19 before or during pregnancy fell ill on 29^th^ December 2021 (at 20^+6^ weeks of gestation) with malaise and symptoms of common cold, running nose and headache, respectively. She tested positive for SARS-CoV-2 on 31^st^ December 2021. On 4th January was admitted to the Division of Gynaecology and Obstetrics, University Medical Center Ljubljana due to hypoxemia and shortness of breath. At admission, the patient was tachypnoeic 28 breaths/minute and hemodynamically stable. She was 21^+5^ weeks pregnant with spontaneously conceived dichorionic twins. This was her second pregnancy. The previous pregnancy was uneventful, she gave birth vaginally, at term, to an appropriate-for-gestational-age weight neonate. She had no complications until the 21st week of gestation. She required oxygen supplementation (35 %) through Venturi mask. Chest radiography showed bilateral peripheral opacities consistent with COVID-19 pneumonia. We started dexamethasone 6 mg qd and prophylactic dose of dalteparin. Due to progression of respiratory failure the patient was transferred to intensive care unit (ICU) of the Department of Infectious Diseases on 6^th^ January 2022. Baseline laboratory values are presented in Table 1.

**Table 1:** Baseline laboratory values on admission day to Intensive Care Unit.

DAY	Reference value	0
**DATE**		6.1.2022
CRP (mg/L)	<5	106
PCT (μg/L)	<0.24	0.20
LYM (x10^9^/L)	1.10	0.20
NEU (x10^9^/L)	1.5–4.00	6.40
NEU/LYM ratio	NA	32.0
IL-6 (ng/L)	<7	45.0
Ferritin (μg/L)	5–204	83
D-dimer (μg/L)	<500	1060

CRP – C-reactive protein, PCT – procalcitonin, LYM – lymphocytes, NEU – neutrophils, IL-6 – interleukin 6, NA - not applicable.

On admission to the ICU, she was tachypnoeic 35 breaths/minute, dyspnoeic while receiving oxygen supplementation via OHIO mask, p02 11,7 kPa, but was hemodynamically stable. Auscultatory breathing was less audible on the right basal side with fine inspiratory cracles on the left side. Initially, we introduced oxygen supplementation through a high-flow nasal cannula (HFNC) (flow 30 L/min; 60 % oxygen). In the following hours she needed an increasingly higher % of added oxygen up to 100 % reaching p02 of 8.80 kPa. She was intubated and mechanically ventilated (MV). Due to high oxygen requirement (Fi02 1.0), inhaled nitric oxide was added and patient was partially therapeutically pronated. Because of rapid respiratory worsening despite treatment with steroids, tocilizumab was added in a single dose (640 mg iv., 8 mg/kg of pre-pregnancy body weight) and dexamethasone changed to methylprednisolone 80 mg qd iv. with slow taper in the next four weeks. SARS-CoV-2 serology came back negative and Delta variant was confirmed, thus casirivimab/imdevimab (single dose 2400 mg iv.) was added on January 7th. Due to the increase in IL-6 empirical antibiotic therapy with piperacillin/tazobactam was started on the same day. The next day thrombosis at the site of the central venous catheter in the right jugular vein was detected sonographically and the dose of dalteparin increased to the therapeutic level. We added flucloxacillin on 10th January, because tracheal aspirate culture grew *Staphylococcus aureus*. We observed erythema and maceration of the skin with marginal peeling in the inguinal and gluteal region. Culture of skin swab yielded *Candida glabrata* and *Enterococcus faecalis*, thus we started topical therapy with fusidic acid, clotrimazole and a corticosteroid cream.

**Fig. 1:**
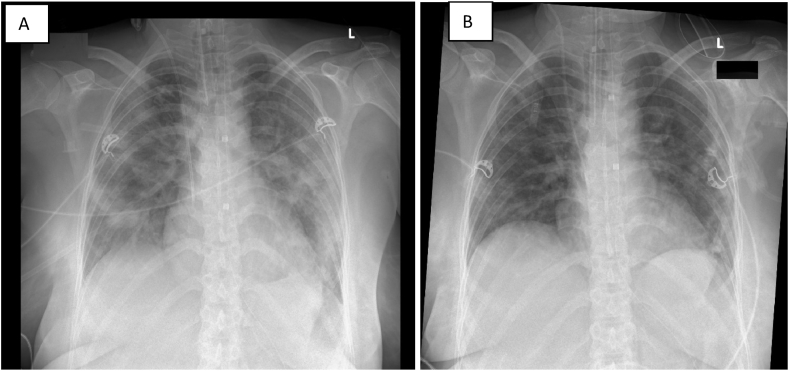
Chest x-rays: A: on 7.1.2022: after orotracheal intubation; B: on 16.1.2022 before extubation.

Clinical and radiologic improvement was observed on 12th of January, and the patient was extubated four days later. Fig. 1 A chest x-ray on 7.1.2022 upon admission and after orotracheal intubation and B on 16.1.2022 before extubation.

During the hospital stay in ICU we did not notice any known or possible adverse reactions to the drugs we used. Fetal surveillance with regular ultrasound examination was performed. Due to selective fetal growth restriction of the twin B, a planned caesarean section was performed at 34^+6^ weeks. Twin A's birth weight was 2820g (male, Apgar 9/9, pH in the umbilical artery 7.31) and twin B's weight was 1775g (male, Apgar 9/9, pH in the umbilical artery 7.30). No congenital anomalies were seen in any of the twins. Twin A was large-for-gestational age and required invasive respiratory support due to neonatal distress syndrome. Twin B was small-for-gestational age and had no respiratory or other complications during the neonatal period [7]. Both twins had no ultrasonographic signs of intraventricular hemorrhage or periventricular leukomalacia at hospital discharge. During the hospital stay we did not notice any known or possible adverse reactions to the drugs we used. After discharge they were followed like other infants for regular systematic examinations. There were no maternal postpartum complications.

## 2 Discussion

We describe a case of successful treatment of a pregnant patient with critical COVID-19 in the second trimester of twin pregnancy who was treated with a combination of corticosteroids, tocilizumab and casirivimab/imdevimab. Corticosteroids were shown to reduce mortality and are considered SoC in treatment of hypoxemic COVID-19 pneumonia [4]. Addition of tocilizumab to corticosteroids in rapid worsening of severe or critical covid-19 was shown to improve survival and shorten the time to clinical improvement [5]. There are very limited data on potential adverse fetal effects of tocilizumab treatment during pregnancy. Analysis of pregnant women exposure to tocilizumab in the second and third trimester has shown an increased rate of prematurity and lower birth weight, which could be attributed to underlying comorbidity, as well [8]. FDA Adverse Event Reporting System (FAERS) from 2020 to 2022 reported 3 safety signals in pregnant women, including neonatal death, which suggests the need of further clinical studies [9]. Except for the USA and Belgium, most other European countries, as well as India and Australia, at least conditionaly recommed the use of tocilizumab in pregnancy [10].

Selective fetal growth restriction seen in of one of the twins occurs in up to 15 % of all dichorionic twin pregnancies [11] and cannot be attributed to tocilizumab treatment with certainty. Moreover, critical maternal illness and steroid therapy can both be associated with impaired fetal growth [12]. We did not observe any other adverse effects on fetal development during follow-up. Our patient received casirivimab/imdevimab as a rescue treatment, as well. Delayed production of neutralizing antibodies has been associated with a more severe course of COVID-19 [13]. Furthermore, treatment of seronegative patients with casirivimab/imdevimab reduced 28-day mortality [14]. Casirivimab/imdevimab in pregnant women with mild-to-moderate COVID-19 decreases the risk of severe disease [6]. Retrospective analysis of pregnant women, who did not have hypoxemia, revealed no difference in obstetrical outcomes compared to controls [15]. Recent meta-analysis shows that casirivimab/imdevimab, reduced the number of cesarean sections, but demonstrated no effect on disease progression and other obstetric and COVID-19 related outcomes [16].

Clinical course was complicated by bacterial infection. It is estimated that more than a third of ICU patients have at least one episode of bacterial infection during hospitalization. Increased risk of secondary infections has been observed with concomitant use of corticosteroids and tocilizumab in COVID-19 [17]. Detection of secondary bacterial infection is often difficult, because combination of dexamethasone and tocilizumab has been shown to reduce inflammatory biomarkers CRP and PCT [18].

## 3 Conclusions

To the best of our knowledge our case presents the first reported use of combination of tocilizumab and monoclonal antibodies casirivimab/imdevimab for critical COVID-19 in twin pregnancy. Our reported clinical case indicates that tocilizumab and monoclonal antibodies casirivimab/imdevimab use during the pregnancy might be safe with favorable outcome, as no severe or minor signs or symptoms in the case presentation were observed neither in the mother nor in infants during the time of observation, but we can't be sure of the possible long term adverse effects of those drugs. Further studies are necessary to confirm safety of this combination treatment in critical COVID-19 of pregnant patients. Without achieving an improvement in the patient's respiratory condition, the pregnancy would have to be terminated in 22nd gestational week, which would certainly mean the inability of the unborn twins to survive. In such cases it is necessary to make an individual judgment on the use of certain drugs and weigh the risks and benefits of its use. Today we are faced with new variants of the virus, such as Omicron, and subvariants that are less virulent and more contagious than Delta. However, we cannot know with certainty that Omicron does not increase the risk of severe maternal morbidity. Therefore, we must be careful, and it is important to know all the tools to manage the disease.

## Authors declare no conflict of interest

Authors received no funds.

A patient's written consent was obtained to publish this report.

Data associated with our study has not been deposited into a publicly available repository. Data will be made available on request.

## CRediT authorship contribution statement

**Nina Grasselli Kmet:** Writing – original draft, Project administration, Investigation, Formal analysis, Data curation, Conceptualization. **Miha Lučovnik:** Writing – original draft, Supervision, Data curation, Conceptualization. **Matjaž Jereb:** Supervision. **Kristina Nadrah:** Writing – original draft, Supervision, Investigation, Formal analysis, Data curation, Conceptualization.

## Declaration of competing interest

The authors declare that they have no known competing financial interests or personal relationships that could have appeared to influence the work reported in this paper.
